# Selenium and Coenzyme Q_10_ Intervention Prevents Telomere Attrition, with Association to Reduced Cardiovascular Mortality—Sub-Study of a Randomized Clinical Trial

**DOI:** 10.3390/nu14163346

**Published:** 2022-08-15

**Authors:** Trine Baur Opstad, Jan Alexander, Jan O. Aaseth, Anders Larsson, Ingebjørg Seljeflot, Urban Alehagen

**Affiliations:** 1 Centre for Clinical Heart Research, Department of Cardiology, Oslo University Hospital Ullevål, 0450 Oslo, Norway; 2 Faculty of Medicine, University of Oslo, 0315 Oslo, Norway; 3 Norwegian Institute of Public Health, 0213 Oslo, Norway; 4 Department of Research, Innlandet Hospital Trust, 2381 Brumunddal, Norway; 5 Faculty of Health and Social Sciences, Inland Norway University of Applied Sciences, 2624 Lillehammer, Norway; 6 Department of Medical Sciences, Uppsala University, 752 36 Uppsala, Sweden; 7 Division of Cardiovascular Medicine, Department of Medical and Health Sciences, Linköping University, 581 83 Linköping, Sweden

**Keywords:** selenium, ubiquinone, telomere, cardiovascular diseases

## Abstract

Short telomeres have been associated with ageing and cardiovascular disease. The influence on leukocyte telomere length (LTL) of long-term intervention with combined selenium and coenzyme Q_10_ is unknown. Our aim was to determine whether 42 months of selenium and coenzyme Q_10_ supplementation prevented telomere attrition and further cardiovascular mortality. The investigation is an explorative sub-study of a double-blind, placebo-controlled, randomized trial. Swedish citizens low in selenium (*n* = 118), aged 70–80 years, were included. Intervention time was 4 years, with 10 years’ follow-up time. LTL was relatively quantified with PCR at baseline and after 42 months. At baseline, LTL (SD) was 0.954 (0.260) in the active treatment group and 1.018 (0.317) in the placebo group (*p* = 0.23). At 42 months, less shortening of LTL was observed after active treatment compared with placebo (+0.019 vs. −0.129, respectively, *p* = 0.02), with a significant difference in change basing the analysis on individual changes in LTL (*p* < 0.001). Subjects suffering future death presented with significantly shorter LTL at 42 months than survivors [0.791 (0.190) vs. 0.941 (0.279), *p* = 0.01], with a significant difference in change of LTL according to cardiovascular mortality and survival (*p* = 0.03). To conclude, preservation of LTL after selenium and coenzyme Q_10_ supplementation associated with reduced cardiovascular mortality.

## 1. Introduction

Ageing is an inevitable process affecting all living cells, with the ensuing accumulation of cellular senescence in organs and tissues and an increased incidence of pathological conditions such as cardiovascular diseases, neurological disorders and cancer [1,2]. The initial mechanisms of ageing are partly mediated by excessive production of reactive oxygen species (ROS) or decreased ROS scavenging, which leads to intracellular oxidative stress [3]. The antioxidant defence in the body includes, among others, selenoproteins comprising the trace metalloid selenium (Se) as the catalytic centre, and certain vitamins. In humans, 25 genes encode selenoproteins. Among these are glutathione peroxidases (GPXs), thioredoxin reductases (TXNRDs) and selenoprotein P, the latter a Se-carrier in plasma, all with anti-oxidative effects potentially combating processes leading to ageing [4].

Telomeres cap chromosome ends and protect DNA and internal genes, preserving chromosomal stability [5]. However, with age, genomic instability occurs partly due to telomere erosion and DNA damage. Telomere attrition over time leads to critically short telomeres and subsequent replicative senescence and organismal ageing [6]. Telomere attrition has also evidentially been linked to coronary artery disease [7]. Oxidative stress and inflammation are major contributors to telomere erosion during cellular replication [8] and are central players in the pathogenesis of atherosclerosis. Se may prevent telomere shortening by anti-oxidative and anti-inflammatory mechanisms, and in vitro studies have reported increased telomerase expression and longer telomeres with Se supplementation [9]; however, evidential clinical research in vivo is lacking.

Another potential modifier of oxidative stress is coenzyme Q_10_ (ubiquinone), primarily present in the mitochondria as a component of the electron transport chain. Se and coenzyme Q_10_ are interrelated, as the cytosolic selenoprotein TXNRD1 reduces ubiquinone to its active antioxidant form ubiquinol [10] and the syntheses of coenzyme Q_10_ and selenoproteins are both dependent on a functional mevalonate pathway [11]. Any impact of coenzyme Q_10_ on telomere shortening has, to our knowledge, not previously been reported.

Because Se intake is low in Europe, and endogenous coenzyme Q_10_ production declines after adolescence, supplementation is thought to restore an imbalance due to low antioxidant status, which otherwise may result in increased cardiovascular (CV) risk, among other effects. We have previously reported beneficial effects of combined supplementation with Se and coenzyme Q_10_ on CV mortality and biomarkers related to inflammation [12,13,14], oxidative stress [15], endothelial dysfunction [16], and microRNA profiling [17].

Leukocyte telomere length (LTL) is thought to reflect telomere length in other cells and tissues, including vascular cells [18,19]. Although short LTL has been linked to CV diseases and all-cause mortality, studies relating longitudinal changes in LTL to CV mortality are scarce [6]. This sub-study’s aim was to explore the impact of long-term supplementation with combined Se and coenzyme Q_10_ on LTL preservation in an ageing population low in Se, with emphasis on LTL’s possible impact on CV mortality.

## 2. Material and Methods

### 2.1. Study Population

**Figure 1:**
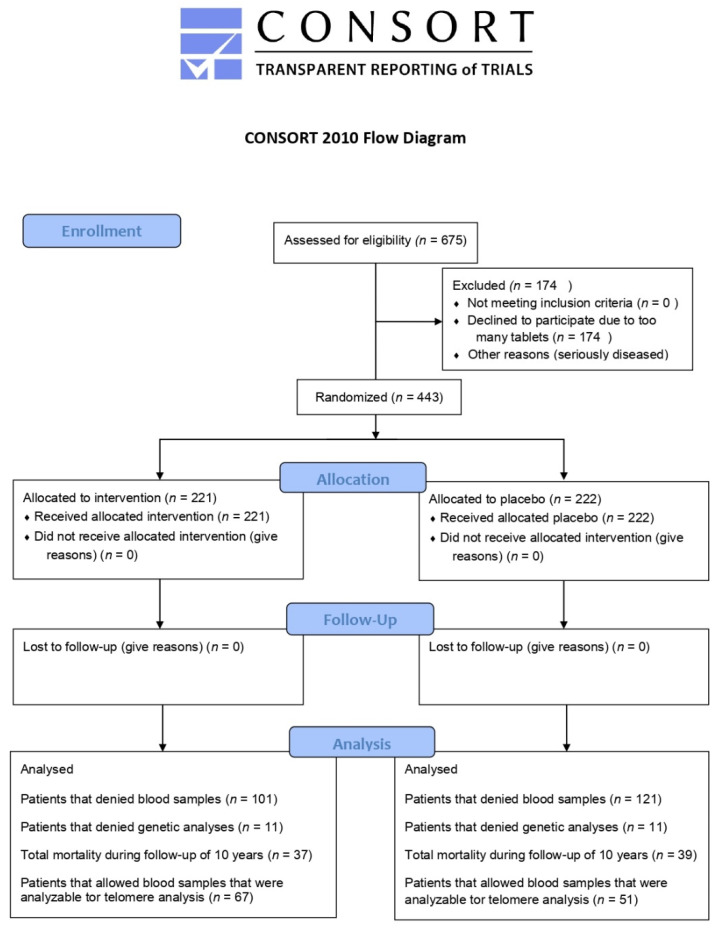
Flow diagram of the selected individuals.

The present investigation is a sub-study of a previous prospective, randomized, placebo-controlled, single-centre trial performed between 2003 and 2010 in the southeast of Sweden [20]. In short, 675 elderly subjects were recruited from a rural municipality of 10,300 inhabitants. Criteria for inclusion were: living in a specific municipality and being aged > 69 years. All persons fulfilling these criteria were invited to participate in the epidemiological project. Exclusion criteria for the main project were: recent myocardial infarction (MI), planned CV operative procedure within 4 weeks, serious disease that substantially reduced survival or when it was not expected that the participant could cooperate for the full 4-year period [20]. Of the 443 included subjects, 219 received the active supplement and 222 participants received a placebo for 4 years. The participants were given either a combination of supplements as 200 µg Se/day of organic Se yeast tablets (SelenoPrecise 100 µg, Pharma Nord ApS, Vejle, Denmark, twice daily) and 200 mg/day of coenzyme Q_10_ capsules (Bio-Quinon 100 mg twice daily, Pharma Nord ApS, Vejle, Denmark), or placebo tablets/capsules. The SelenoPrecise^®^ 100 µg tablet is approved in Denmark as a pharmaceutical drug by the Danish Medicines Agency and the Q_10_ capsules were identical to Myoqinon^®^ (Pharma Nord Aps, Vejle, Denmark), which is a pharmaceutical drug authorized in European Union Member State (No. OGYI 11494-2010). The placebo tablets of Se consisted of baker’s yeast only. The placebo capsules of the coenzyme Q_10_ contained 500 mg of a vegetable oil that was added with 3.1 mg vitamin E. All participants were supplemented for 48 months, and non-consumed study medication was returned and counted as a measure of compliance. In the present sub-study, we used blood samples retrieved at inclusion and at 42 months. Individuals who refused to give blood samples for DNA analyses or chose to withdraw from sub-studies or died during the intervention time (*n* = 325) were not included in the present investigation. Therefore, the final population of municipality living elderly persons comprised 118 subjects, of whom 67 were on active treatment and 51 received placebo (Figure 1, Flowchart).

One of three experienced cardiologists examined all study participants at inclusion. A new clinical history, a clinical examination and assessment of New York Heart Association functional class (NYHA class), as well as an electrocardiogram (ECG) and Doppler-echocardiography were performed at inclusion. The ejection fraction (EF) readings were categorized into four classes, with interclass limits at 30%, 40% and 50% and with normal systolic function defined as EF≥ 50%. Severely impaired systolic function was defined as EF < 30%. CV mortality was recorded for all study participants over a period of 6 years following completion of the study. Information according to mortality was obtained from the National Board of Health and Welfare in Sweden, registering all deaths of Swedish citizens based on death certificates or autopsy reports. The definition of CV mortality was mortality due to MI, cerebrovascular lesions, fatal cardiac arrhythmias, heart failure or aortic aneurysms.

The intervention study conformed to the Declaration of Helsinki and was approved by the Regional Ethical Committee in Sweden (Diary no. 03-176). Written informed consent was obtained from all participants and the study is registered at Clinicaltrials.gov, with identification number NCT01443780.

### 2.2. Biochemical Analyses

Blood samples were collected under fasting conditions at inclusion and after 42 months. Routine analyses were carried out by conventional methods. Pre-chilled ethylenediaminetetraacetic acid (EDTA) vials were centrifuged at 3000× *g* at +4 °C, and plasma was frozen at −70 °C for measurement of Se concentration by inductively coupled plasma mass spectrometry (ICP-MS) [21]. EDTA whole-blood for telomere length analysis in circulating leukocytes was kept frozen at −70 °C until further preparation. Samples for DNA isolation were available for all 118 participants before and after intervention.

### 2.3. DNA Extraction

DNA was isolated manually by the QIAamp DNA Blood Mini Kit, with the same lot number throughout the study (Qiagen GmbH, Hilden, Germany). DNA purity and quantity were tested on the NanoDrop, ND-1000 (Saveen Werner, Sweden). DNA was successfully extracted from all samples and was stored at −80 °C. The achieved mean DNA concentration was 38 ng/µL.

### 2.4. LTL Determination

Equal amounts of extracted DNA per experiment (final concentration 2 ng/μL) were used to measure LTL by singleplex quantitative real-time polymerase chain reaction (PCR) [22]. PCR amplification was performed on the VIIa^TM^7 instrument (Applied Biosystems by Life Technologies, Foster City, CA, USA), using telomere-specific primers (Invitrogen by Thermo Fisher Scientific, Waltham, MA, USA) (Supplementary Table S1) and GoTaq^®^qPCR Master Mix (Promega, Madison, WI, USA). LTLs were relatively quantified to the single-copy gene (SCG) SB34 (Invitrogen by Thermo Fisher Scientific) (Supplementary Table S1) and an internal reference sample. The primers for both targets were diluted to a final concentration of 4 pmol /µL. PCR conditions for both targets were as follows: an initial step at 95 °C for 10 min followed by 40 cycles of 95 °C for 15 s and 60 °C for 1 min. A template negative control was included for both assays in each run to exclude contamination, and all samples were run in triplicate. Individual amplification curves for all samples of both assays were carefully validated, totalling 1416 curves. Technical triplicates with an SD exceeding 0.5 Ct were excluded from the analysis, with two remaining valuable parallels. LTLs were successfully analyzed in all available samples (*n* = 236), with 50 samples reanalyzed. Samples from the intervention and placebo groups were run simultaneously on the same PCR plate.

### 2.5. Statistical Methods

Descriptive data are presented as percentages or mean ± standard deviation (SD). A Student’s unpaired two-sided *t* test was used for continuous variables and the chi-square test was used for analysis of one discrete variable. As the dataset demonstrated a slight non-Gaussian distribution, the dataset was log-transformed when evaluating continuous variables to obtain a normal distribution. The effect of this transformation was controlled through a Kolmogorov–Smirnov test. Transformed data were used in the *t*-test evaluations. All evaluations were performed according to the intention-to-treat principle. Repeated measures of variance were used in order to obtain individual changes in the length of the telomeres. Both transformed and non-transformed data were applied in the analysis of covariance (ANCOVA) evaluation, with no significant difference in the results. In the multivariable model, LTL after 42 months was used as the dependent variable and the allocated interventions as one of the independent variables. Adjustments were made for sex and age by convention, whereas smoking, hypertension, diabetes, ischaemic heart disease (IHD), C-reactive protein (CRP), NYHA class III and LTL at inclusion were added in the model based on relevance for either telomere attrition and/or clinical relevance for CV mortality. A Kaplan–Meier curve was generated in which the first quartile of telomere shortening was included as reference against the three upper quartiles, with regard to CV mortality within 10 years after inclusion. *p*-Values < 0.05 were considered statistically significant, based on a two-sided evaluation. All data were analyzed using standard software (Statistica v. 13.2, Dell Inc., Tulsa, OK, USA).

## 3. Results

### 3.1. Baseline Characteristics

The baseline characteristics of the study population, divided into intervention with active substances or placebo, are shown in Table 1. The mean age of the total population was 77 years and 59% (46 of 118) were females. At inclusion, no difference in clinical characteristics was observed between the randomized groups. No participants presented with NYHA functional class IV and there was a tendency for statistical overweight in numbers of subjects with NYHA class III in the group allocated to active treatment (*p* = 0.05). Plasma Se concentration at inclusion was below the required amounts for the proper expression of selenoproteins [23,24] and did not differ between the groups (mean [SD], 66.5 [15.9] μg/L in the active treatment group and 67.4 [17.2)] μg/L in the placebo group, *p* = 0.56). At baseline, LTL and Se status were weakly but significantly correlated, Spearman’s Rho = 0.2, *p* = 0.01.

### 3.2. Effects of Intervention on Leukocyte Telomere Length

**Figure 2:**
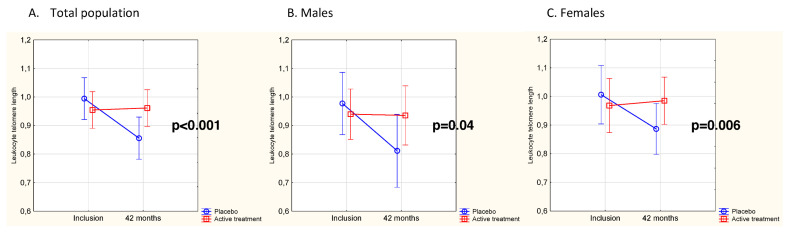
(**A**) LTL at baseline and after 42 months in the Se and coenzyme Q_10_ treatment group compared to the placebo group in the total study population. Evaluation performed by use of repeated measures of variance. Current effect F(1,116) = 11.8, *p* < 0.001. Vertical bars denote 95% CI. The blue line represents the placebo group, the red line the active treatment group. (**B**) LTL in males at baseline and after 42 months in the Se and coenzyme Q_10_ treatment group compared to the placebo group. Evaluation performed by use of repeated measures of variance. Current effect F(1, 51) = 4.63, *p* = 0.04. Vertical bars denote 95% CI. The blue line represents the placebo group, the red line the active treatment group. (**C**) LTL in females at baseline and after 42 months in the Se and coenzyme Q_10_ treatment group compared to the placebo group. Evaluation performed by use of repeated measures of variance. Current effect F(1, 63) = 8.09, *p* = 0.006. Vertical bars denote 95% CI. The blue line represents the placebo group, the red line the active treatment group.

No significant difference was observed between the active treatment group (*n* = 67) and the placebo group (*n* = 51) in LTL values at baseline (mean [SD] LTL, 0.954 [0.260] vs. 1.018 [0.317], respectively, *p* = 0.23). When analyzing the individual changes in LTL from inclusion to 42 months, a significant difference in change of LTL was observed in the active treatment group vs. the placebo group (delta LTL, +0.019 vs. −0.129, *p* = 0.02). Upon validating the obtained results by use of repeated measures of variance, the difference persisted (Figure 2A) (*p* < 0.001). As a second step of validation, a multivariable model was applied and significantly longer LTLs could be demonstrated in the active treatment group also after adjusting for sex, age, smoking, hypertension, diabetes, IHD, CRP, NYHA class III and LTL at inclusion (*p* = 0.03) (Supplementary Table S2). Stratified by sex, the difference in LTL change after 42 months between the randomized groups was statistically significant in both males (*p* = 0.04) and females (*p* = 0.006) (Figure 2B,C), with no significant difference in telomere attrition between sexes (*p* = 0.62), regardless of the intervention used.

### 3.3. Difference in Change of LTL as Related to CV Mortality

**Figure 3:**
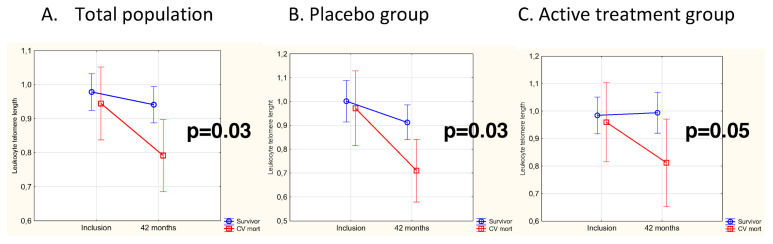
(**A**) LTL in the CV mortality group compared to survivors at baseline and after 42 months in the total population. Evaluation performed by use of repeated measures of variance. Current effect F(1,116) = 4.62, *p* = 0.03. Vertical bars denote 95% CI. The blue line represents the placebo group, the red line the active treatment group. (**B**) LTL in the CV mortality group compared to survivors at baseline and after 42 months in the placebo group. Evaluation performed by use of repeated measures of variance. Current effect F(1,49) = 5.30, *p* = 0.03. Vertical bars denote 95% CI. The blue line represents the placebo group, the red line the active treatment group. (**C**) LTL in the CV mortality group compared to survivors at baseline and after 42 months in the active treatment group. Evaluation performed by use of repeated measures of variance. Current effect F(1,65) = 3.96, *p* = 0.05. Vertical bars denote 95% CI. The blue line represents the placebo group, the red line the active treatment group.

In the period from the completion of the study at 48 months to the end of the following 6 years, 24 CV deaths were registered, 12 (18%) in the active treatment group and 12 (24%) in the placebo group. At inclusion, LTL was not differently distributed between survivors and subjects suffering CV death during the follow-up (mean [SD] LTL, 0.659 [0.510] vs. 0.810 [0.410], *p* = 0.14). In the total population, LTL measured at 42 months was significantly longer in survivors than in the group who suffered CV mortality (mean [SD] LTL, 0.941 [0.279] vs. 0.791 [0.190], *p* = 0.01). For validating the variation in LTL from inclusion to 42 months, the individual change in LTL was evaluated by applying repeated measures of variance, and the significantly longer LTL in the survivor group persisted (*p* = 0.03) (Figure 3A). Upon stratification according to placebo and active treatment and applying repeated measures of variance, significantly shorter LTL in subjects suffering CV mortality was found in the placebo group (*p* = 0.03) (Figure 3B). In those receiving active treatment, a borderline significance was observed, with shorter LTL in the CV mortality group compared with the survivors (*p* = 0.05) (Figure 3C).

**Figure 4:**
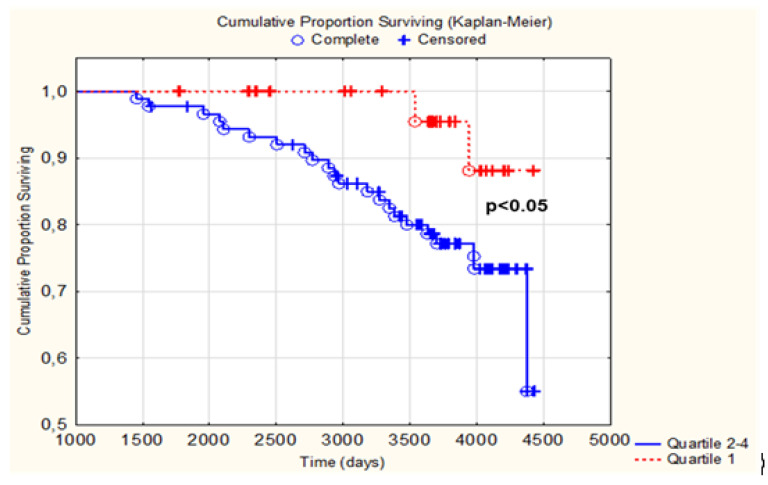
Kaplan–Meier graph illustrating those with a telomere shortening in the 1st quartile vs. quartiles 2–4 regarding CV mortality within 10 years. The red line represents quartile 1 and the blue line represents quartiles 2–4.

The generated Kaplan–Meier curve for the total population shows that subjects in the lowest quartile of telomere shortening survived significantly longer than subjects in the three upper quartiles (*p* < 0.05) (Figure 4).

## 4. Discussion

The main finding in our study was that supplementation with combined Se and coenzyme Q_10_ for 42 months prevented telomere attrition in an elderly Swedish population low in Se. Those who died a CV death during 6 years of follow-up after completion of the study at 48 months presented with shorter LTL as measured at 42 months compared to survivors. Less telomere shortening during the follow-up period was associated with significantly longer survival. No significant sex differences were noted.

To the best of our knowledge, this is the first population-based study measuring the effect of long-term intervention with combined Se and coenzyme Q_10_ on LTL. The beneficial influence on telomere length was observed in both sexes, although a slightly stronger impact on telomere length preservation might be suggested in females. This effect could potentially be due to initial lower levels of coenzyme Q_10_ and its active form ubiquinol, which previously has been reported in women [25]. Our finding of a significant positive correlation between LTL and plasma Se concentration at baseline concurs with a recent cross-sectional observational study reporting that dietary Se intake was related to longer telomeres in middle-aged and elderly Americans [26]. In mice, dietary Se deprivation was observed to induce telomere shortening in colonocytes carrying humanized telomeres [27]. These reports illustrate the impact of Se on telomere length preservation and substantiates the results of our study performed in subjects living in an area with low selenium content in the soil. Little is known about the effects on telomeres of coenzyme Q_10_ in monotherapy. However, in an early in vitro study, less telomere shortening was observed in fibroblasts when exposing the cells to 10-(6′-ubiquinonyl) decyltriphenylphosphonium bromide, a ubiquinone derivative that selectively accumulates in mitochondria and blocks oxidative damage [28].

We also observed shorter LTL at 42 months in the study participants that later suffered CV death compared with survivors up to 6 years after completion of the study. Although a causal link to the intervention is not proved from our study, the results indicate a possible association between telomere length and CV mortality. We also found a significantly higher survival rate in subjects with less telomere shortening (Figure 4), which, to our knowledge, has not previously been reported, and even with the small size of our study, the results were clear. Our results accord with prospective and observational studies showing associations between telomere length and ischaemic heart diseases, indicating a possible link between telomere attrition and increased CV risk [6,29,30]. These data are somewhat in contrast to findings in the prospective Cardiovascular Health Study, including subjects at similar age as in our population, reporting shorter LTL to be significantly associated with death caused by infection diseases but only borderline significantly associated with CV death [31]. Similarly, in two population-based prospective cohort studies, including subjects younger than ours (age range 43–75 years), LTL predicted all-cause mortality, with no significant association with CV mortality [32]. However, in three recently reported longitudinal studies, LTL associated with all-cause mortality, with the strongest association to CV death, compared to cancer and other causes [33]. Notably, and in light of these findings, we are the first to report beneficial effects of long-term supplementation with Se and coenzyme Q10 on LTL preservation with association to CV outcome.

The observed borderline significance regarding CV mortality in the active treatment group was probably due to the limited study sample. It is also interesting to note a visual difference in graph steepness (Figure 3), representing LTL changes between those who experienced a CV death and those who did not. The less pronounced difference in the active treatment group could also be a result of anti-inflammatory and/or anti-oxidative effects of the supplementation, resulting in a less diseased population also in those who died a CV death. Whether the observed CV protection is also mediated via the preservation of telomeres is questionable. However, our results indicate that the general loss in telomere length with age was probably retarded or decelerated during the intervention period. Although the study participants undoubtedly had been exposed to CV risk factors for many years or perhaps life-long, the intervention has improved their phenotype, here presented as preserved telomeres, with possibly less cellular damage. As cellular stress due to low-grade inflammation and oxidative processes may affect both telomere instability and the pathogenesis and progression of atherosclerosis [34], the intervention may have been protective of both mechanisms.

The associations between Se/coenzyme Q_10_ and LTL observed in the present study can be mediated via several mechanisms. It is well accepted that several selenoproteins and coenzyme Q_10_ are strong cellular red/ox regulators and antioxidants with protective effects on biomacromolecules, such as proteins and nucleic acids. The production of ROS increases with age, which makes improvement of the antioxidant defence more important in the elderly to prevent age-related diseases. TXNRD1 and particularly selenoprotein H, highly expressed in the nucleus, protect against oxidative stress and damage to DNA and play a role in gene maintenance, telomere length and function [35]. The beneficial effect of Se on telomere length may also be mediated by the expression of sirtuins, which are important intracellular molecules involved in DNA repair and the maintenance of genomic stability and longevity [36].

Our previous published findings on the beneficial effects of combined supplementation with Se and coenzyme Q_10_ on CV mortality [14,20,37] inflammation, oxidative stress and endothelial dysfunction [12,13,15,16], along with the present preventive effect on telomere attrition and indirectly CV mortality risk, underline the anti-inflammatory, anti-oxidative, and anti-ageing effects of such intervention in the elderly low in Se.

Although statistically significant results were achieved, the sample size was limited, and results should be interpreted with caution and regarded as hypothesis generating. As our population was homogenous, consisting only of Caucasians, the results cannot be generalized to other ethnicities. Additionally, the inclusion of indicative “healthy” elderly can be discussed as an incorrect term, as some of the included subjects had mild to moderate heart failure symptoms or diabetes. Nevertheless, this might also have strengthened the study, as it reflects the general population of similar age living in society. There were no selection restrictions besides living in the specific municipality and having a specific age interval to characterize the study population. A limitation might be that blood samples for DNA analysis were retrieved at 42 months and not at the study follow-up at 48 months. Nevertheless, accomplishing nearly 90% of the intervention time, we find it unlikely that another 6 months would have changed the results. Although LTL is presumed to reflect telomere length in vascular cells [18], it may not necessarily mirror alteration in the myocardium. Nevertheless, telomere shortening has been associated with endothelial senescence accelerated by oxidative stress [38], which corresponds well with our results that Se, with its anti-oxidative properties, has a protective function. The narrow age span in the studied cohort may also have influenced the data, as telomere attrition generally declines in older age [29,30]. Moreover, the differentiated effect of Se vs. coenzyme Q_10_ on LTL cannot be addressed by this study. However, the results are strengthened by the long intervention time and the extended follow-up period for another 6 years for the recording of CV mortality. A two-step statistical evaluation strengthens the validity of the results.

## 5. Conclusions

Supplementation with Se and coenzyme Q_10_ combined prevented significantly leukocyte telomere attrition in elderly people low in Se, which shows the importance of anti-inflammatory and antioxidant mechanisms in the prevention of ageing. Although causality in the intervention is not proven by our exploratory study, the observed preservation of telomeres along with longer survival was clear, indicating the telomeres’ preventive contribution in the reduction of CV mortality.

## Figures and Tables

**Table 1:** Baseline characteristics of the study population receiving Se and coenzyme Q_10_ supplementation or placebo.

	Active Treatment Group *n* = 67	Placebo Group *n* = 51
Age, median (range)	77 (14)	77 (15)
Females 65/Males 53	35/32	30/21
History		
Smoking, *n* (%)	7 (10)	6 (12)
Diabetes, *n* (%)	10 (15)	9 (18)
Hypertension, *n* (%)	46 (69)	40 (78)
Hb, mean (SD), g/L	137 (19.5)	136 (12.6)
IHD, *n* (%)	17 (25)	8 (16)
NYHA class I, *n* (%)	36 (54)	32 (63)
NYHA class II, *n* (%)	17 (25)	14 (28)
NYHA class III, *n* (%)	14 (21)	4 (8)
NYHA class IV, *n*	0	0
Unclassified, *n*	0	1
Medications		
Anticoagulants, *n* (%)	5 (8)	5 (10)
ACEI/ARB, *n* (%)	8 (12)	10 (20)
Beta-blockers, *n* (%)	25 (37)	19 (37)
Diuretics, *n* (%)	22 (33)	17 (33)
Statins, *n* (%)	14 (21)	11 (22)
Se conc. incl. mean (SD), µg/L	66.5 (15.9)	67.4 (17.2)
Se conc. 48 months, mean (SD) µg/L	208.7 (57.0)	71.6 (24.9)
Q10 conc. incl. mean (SD mg/L	0.82 (0.31)	0.81 (0.31)
Q10 conc. 48 months, mean (SD) mg/L	2.19 (1.35)	0.90 (0.36)
Examinations		
EF < 40%, *n* (%)	5 (7)	2 (4)
Atrial fibrillation, *n* (%)	3 (5)	3 (6)
Mortality		
CV death, *n* (%)	12 (18)	12 (24)

SD, standard deviation; IHD, ischaemic heart disease; NYHA, New York Heart Association functional class; CV, cardiovascular; ACEI, angiotensin-converting enzyme inhibitor; ARB, angiotensin.

## Data Availability

The data presented in this study are available on request from the corresponding author. The data are not publicly available due to Swedish Law. The authors cannot share the data used in this study and cannot conduct any further research other than what is specified in the ethical permissions application. For inquiries about the data, researchers should first contact the owner of the database, the University of Linköping. Please contact the corresponding author with requests for and assistance with data. If the university approves the request, researchers can submit an application to the Regional Ethical Review Board for the specific research question that the researcher wants to examine.

## Supplementary Materials

The following supporting information can be downloaded at: https://www.mdpi.com/article/10.3390/nu14163346/s1, **Table S1**: Nucleotide sequence for the telomere and single-copy gene analyses **Table S2**. Analysis of covariance using leukocyte telomere length at 42 months as dependent variable.
