# Supplementary material for: Selenium and Coenzyme Q10 Intervention Prevents Telomere Attrition, with Association to Reduced Cardiovascular Mortality—Sub-Study of a Randomized Clinical Trial
Source: Nutrients. 2022 Aug 15;14(16):3346. doi: 10.3390/nu14163346 (PMC9412367; doi:10.3390/nu14163346)
Supplement: Supplementary file 1 [file nutrients-14-03346-s001.zip › nutrients-1816478-supplementary.pdf]

Supplementary material

Supplementary Table S1: Nucleotide sequence for the telomere and single copy gene analyses

Supplementary Table S2. Analysis of covariance using leukocyte telomere length at 42 months as dependent variable

Supplementary Table S1 Nucleotide sequence for the telomere and single copy gene analyses

PCR primers	Oligomer sequences (5’ to 3’)
Telomere fw	CGG TTT GTT TGG GTT TGG GTT TGG GTT TGG GTT TGG GTT
Telomere rev	GGC TTG CCT TAC CCT TAC CCT TAC CCT TAC CCT TAC CCT
Single copy gene (36B4) fw	CAG CAA GTG GGA AGG TGT AAT CC
Single copy gene (36B4) rev	CCC ATT CTA TCA TCA ACG GGT ACA A

Fw; forward, rev; reverse

<u>Supplementary Table S2</u> Analysis of covariance using leukocyte telomere length at 42 months as dependent variable

Effects	Mean Squares	Degrees of freedom	F	P
Intercept		0		
Gender	0.013	1	0.22	.64
Age	0.009	1	0.15	.70
Smoking	0.126	1	2.05	.16
Hypertension	0.120	1	1.96	.17
Diabetes	0.062	1	1.00	.32
IHD	0.04	1	0.70	.41
HsCRP at inclusion	0.003	1	0.05	.82
NYHA class III	0.003	1	0.04	.83
Active treatment	0.301	1	5.03	.03
Telomere length at inclusion	0.748	1	12.17	.001
Error	0.06	43		

IHD; Ischaemic heart disease, HsCRP; High sensitivity C-reactive protein, NYHA; New York Heart Association functional class III
